# The Enzymatic Synthesis of Perdeuterated D- and L-Lactic Acid-*d*_4_ and Polymerization of Their Lactides to Polylactic Acid

**DOI:** 10.3390/bioengineering12060575

**Published:** 2025-05-27

**Authors:** Anna E. Leung, Andreas Raba, Klaus Beckerle, Jürgen Allgaier, Hanna P. Wacklin-Knecht

**Affiliations:** 1 European Spallation Source ERIC, P.O. Box 176, 221 00 Lund, Sweden; 2 Jülich Centre for Neutron Science (JCNS-1), Forschungszentrum Jülich GmbH, Leo Brandt Straße, 52425 Jülich, Germany; 3 Institute for Inorganic Chemistry, RWTH Aachen University, 52074 Aachen, Germany; 4 Division of Physical Chemistry, Department of Chemistry, Lund University, 221 00 Lund, Sweden

**Keywords:** deuteration, biopolymers, synthesis, polylactic acid, enzymatic

## Abstract

We report the synthesis of highly enantiopure perdeuterated poly-L-lactic acid and poly-D-lactic acid polymers with well-defined molecular weight by polymerization of perdeuterated lactides. Enantiopure D- and L-lactic acid-*d*_4_ monomers were synthesized from sodium pyruvate-*d*_3_ using D- and L-lactate dehydrogenase enzymes (D-LDH and L-LDH) as biocatalysts. The reduced form of the co-enzyme nicotinamide adenine dinucleotide-*d*_1_ (NADH-*d*_1_) was generated in situ from the oxidized form nicotinamide adenine dinucleotide (NAD^+^) by formate dehydrogenase (FDH)-catalyzed oxidation of sodium formate-*d*_1_ to carbon dioxide with concerted reduction of NAD^+^ to NADH-*d*_1_. For the conversion of the perdeuterated lactic acid monomers to the corresponding lactide dimers, we developed a process for generating these compounds in the high purity needed for the final anionic ring-opening polymerization step. This method enabled the generation of a range of perdeuterated polylactic acid polymers that are highly suitable for the characterization of polymer structure and dynamics using neutron scattering, infrared and nuclear magnetic resonance spectroscopy methods that are sensitive to deuterium. Furthermore, these deuterium-labeled polymers are well-suited to the study of the biodegradation of PLA-based plastics.

## 1. Introduction

Polylactic acid (PLA) polymers are an attractive alternative to conventional, petrochemical-based polymers due to their biodegradability and sustainability. They are widely used for packaging materials, disposable tableware, agricultural plastics and are ideally suited for 3D printing applications. Lactic acid, also widely used by the pharmaceutical and food industries, is chiral, and its polymerization results in isotactic, syndiotactic or atactic PLA depending on the chirality of the monomers, which also leads to different physical properties and biodegradability [1]. In addition, stereo-complexation [2] between the two PLA enantiomers, poly-L-lactic acid (L-PLA) and poly-D-lactic acid (D-PLA), leads to significant improvements in material properties, such as increased thermal stability, and presents possibilities for directed self-assembly of novel designer materials based on complementary strands. The properties of PLA polymers are highly dependent on molecular weight: this is beyond the scope of this manuscript but has been discussed elsewhere [3].

The design of such materials necessitates the synthesis and characterization of high-quality and well-defined PLA polymers with a narrow molecular weight distribution, and characterization using advanced methods. Neutron scattering offers a particular advantage in elucidating the structure and function of complex polymer materials, as, in combination with deuterium labeling, it can be used to highlight details of the polymer arrangement that would otherwise be undetectable [4]. It is therefore of interest to develop synthetic methods that allow perdeuterated PLA polymers to be generated with a well-defined chirality, which requires starting from enantiopure perdeuterated lactic acid monomers. To the best of our knowledge, the synthesis of the perdeuterated lactic acid enantiomers has not been reported previously.

Perdeuteration of small molecules can be achieved using a variety of methods for inducing hydrogen/deuterium (H/D) exchange reactions at carbon centers [5] including pH-dependent exchange in D_2_O or deuterated protic solvents [6], using deuterated reducing or methylating reagents [7], and hydrothermal metal-catalyzed exchange using D_2_ or D_2_O as deuterium source [8,9]. These methods, however, require that the target molecules contain only C-H moieties which are acidic or which can be installed via deuterated reagents, or that the target molecules are robust to hydrothermal conditions, i.e., without sensitive functionalities or chiral centers [10]. Lactic acid enantiomers are not candidates for these reactions because they possess a chiral center. However, the common, prochiral precursor in their synthesis, sodium pyruvate-*d*_3_, can be produced from pyruvic acid using known methods [6].

Biocatalysis, using purified enzymes, is one alternative to traditional methods of chemical synthesis, and, when applied to the area of deuterium-labeled compounds, facilitates challenging transformations, such as the enantioselective installation of deuterium [11]. The synthetic use of lactate dehydrogenase enzymes (LDH) to produce enantiopure lactate from pyruvate is well established, as are methods for the immobilization of the enzymes [12,13,14,15], and the in situ regeneration of the co-factor, NADH [12,14,15,16,17,18]. Such a system has been reported to produce unlabeled zinc D-lactate from pyruvate [12]. We therefore hypothesized that this method could be used to produce perdeuterated, enantiopure D-lactic acid by starting from sodium pyruvate-*d*_3_ and NADH-*d*_1_, with the latter (re)generated by a second catalytic cycle in which formate dehydrogenase (FDH) oxidizes sodium formate-*d*_1_ to CO_2_. Sodium formate-*d*_1_ is commercially available. Importantly, according to the mechanism of LDH and FDH, the deuterium is transferred via NADH-*d*_1_ to the pyruvate-*d*_3_, and originates from the sodium formate-*d*_1_ [19]. This allows for the synthesis of perdeuterated D-lactate without the need for a deuterated solvent [19,20]. L-lactic acid-*d*_4_ could be produced using the same method, substituting L-LDH for D-LDH.

The production of polylactic acid from lactic acid is usually carried out in a two-step process by first converting the lactic acid into the dimer, lactide, which is then polymerized anionically or by a coordination/insertion mechanism [21]. The polymerization demands lactide of high chemical purity. The established methods for lactide production are based on high temperature oligomerization-depolymerization processes [22], with disadvantages such as partial loss of chirality, formation of non-recoverable material, and low yields, particularly if only small material quantities are processed. Therefore, on the basis of a recently developed procedure using zeolites as shape-selective catalysts [23], we developed a process that allows the direct stereoselective conversion of lactic acid into lactide. The method was optimized to obtain lactide monomers of high purity for subsequent anionic ring-opening polymerization using a readily available catalytic system composed of diethylzinc and benzylic alcohol. Ring-opening polymerization of lactides by ZnEt_2_/ROH catalytic systems has long been established [24,25,26] and shown to be a versatile method [27,28]. While it is neither exceedingly fast nor overly selective, it offers a route to block copolymers if a macroinitiator is employed as the alcohol component [29]. Such block copolymers are of interest to synthesize for investigating their self-assembly into solution structures, such as micelles, using neutron scattering and nuclear magnetic resonance (NMR) spectroscopy methods.

Perdeuterated PLA is not only useful for obtaining a fundamental understanding of its physical properties using neutron scattering experiments. The deuterium label also makes it useful for studying microbial degradation pathways of PLA-based plastics widely used in agriculture. Recently, perdeuterated D-polylactic acid, synthesized according to the method reported here, was used to trace the incorporation of microplastics into microbial cells using Raman microspectroscopy [30].

## 2. Materials and Methods

### 2.1. Chemicals

D-LDH (lyophilized powder from *Lactobacillus leichmanii)*, L-LDH (from rabbit muscle), FDH (from *Candida boidinii),* and NAD^+^ hydrate (from yeast) were purchased from Sigma Aldrich (St. Louis, MO, USA). Sodium formate-*d*_1_ was purchased from Cambridge Isotope Laboratories (Tewksbury, MA, USA). Deuterium oxide (99.8%), pyruvic acid, L-lactic acid, sodium bicarbonate, tris-HCl, NaOH, and HCl were purchased from Sigma Aldrich. All other reagents were commercial products and used as received, except benzyl alcohol which was dried over molecular sieves and degassed prior to use. Tetrahydrofuran (THF) was obtained from a MBraun (Garching, Germany) SPS (solvent purification system). Solvents were used as purchased from Sigma Aldrich or Honeywell (Charlotte, NC, USA). Zeolite Beta (Si/Al 12.5) was provided by Clariant (Heufeld, Germany).

### 2.2. Monomer and Dimer Characterization

High resolution mass spectrometry (HRMS) was performed on a Waters (Milford, MA, USA) quadrupole time-of-flight (QTOF) XEVO-G2. NMR spectroscopy was performed on a Varian (Palo Alto, CA, USA) Unity INOVA spectrometer (^1^H 400 MHz; ^13^C 100 MHz; ^2^H 61 MHz). NMR spectra were referenced to the residual solvent where applicable. Deuterium incorporation was calculated from mass spectra using a comparison of the isotopologues, taking into consideration the contribution of ^13^C species.

Gas chromatography-mass spectrometry (GC-MS) measurements of D- and L-lactic acid-*d*_4_ (as their menthyl ester derivatives, see SI Figures S11 and S12) were performed on a Shimadzu (Kyoto, Japan) GCMS-QP2010 equipped with a TG-5SIL MS column (length: 20 m; I.D.: 0.18 mm; film 0.36 µm). The linear velocity was 36.5 cm/s and the oven program started at 60 °C (hold 2 min) with a heating ramp of 10 °C/min to 290 °C (hold 5 min). A lactic acid sample (11 ± 0.5 mg) was mixed with (−)-menthol (54 ± 1 mg) in a thick-walled glass tube. *para*-Toluenesulfonic acid (1.9 ± 0.2 mg) was added and the mixture was dissolved in mesitylene (150–160 mg) and heated to 110 °C overnight (14 h). A drop of the resulting mixture was taken up in dichloromethane (1.5 mL) and injected into the GC-MS.

Specific rotation was measured using a Jasco (Tokyo, Japan) P-2000 polarimeter in DCM (λ = 589 nm at 23 °C) in a 1.0 dm measuring cell.

### 2.3. Polymer Characterization

NMR spectroscopy was performed on a Bruker (Billerica, MA, USA) DRX 400 spectrometer (^1^H 400.1 MHz, ^13^C 125.6 MHz) at 25 °C. Size exclusion chromatography (SEC) measurements were performed on an Agilent (Santa Clara, CA, USA) 1100 series instrument at ambient temperature with SDV linear M columns (8 mm × 300 mm and 8 mm × 600 mm) purchased from Polymer Standard Service (Agilent), using THF as solvent and polystyrene standards. Differential scanning calorimetry (DSC) was measured on a Netzsch (Selb, Germany) DSC 204 Phoenix in a pierced aluminum crucible at heating and cooling rate of 10 K/min and 5 min isothermal segments between heating and cooling phases. Nitrogen was used as the purge gas at 20 mL/min.

## 3. Synthesis

### 3.1. Sodium Pyruvate-d_3_ (***1***)

Using a modification of a published method [6], pyruvic acid (10.01 g, 113.7 mmol) was dissolved in D_2_O (500 mL, 27.7 mol) in a 3-neck round-bottom flask fitted with a coil condenser and a thermometer. The solution was boiled at 100 °C for 6 h, until ^1^H NMR analysis of aliquots showed that no further H/D exchange was occurring. The solution was cooled to room temperature and sodium bicarbonate (9.07 g, 108.0 mmol, 0.95 equiv) was added. The solution was concentrated under reduced pressure and the resulting solid was recrystallized from a 1:12 mixture of recovered D_2_O:absolute ethanol (65 mL) to afford an off-white solid. A second concentration/recrystallisation of the filtrate afforded a second batch which was combined with the first (total yield 9.05 g, 70%).

### 3.2. D-Lactic Acid-d_4_ (***2***) and L-Lactic Acid-d_4_ (***3***)

A solution of sodium pyruvate-*d*_3_ (10.0 g, 88.5 mmol, 91%D) and sodium formate-*d*_1_ (6.71 g, 97.2 mmol) was prepared in 1 L distilled water containing 5 mM tris-HCl in a cylindrical, flat-bottomed, multi-neck flask with a flat ground flange. The pH was adjusted to 7.5 using 0.1 M NaOH in H_2_O and the system was fitted with a pH probe, a tube for 0.1 M HCl addition, a magnetic spin bar and a needle for argon gas throughput, and de-gassed for 1 h. β-nicotinamide adenine dinucleotide hydrate NAD^+^xH_2_O (593 mg, 894 μmol for D-lactic acid-*d*_4_ synthesis/583 mg, 879 μmol for L-lactic acid-*d*_4_ synthesis) was added and then a 10 mL aliquot of the mixture was removed and added to a mixture of D-LDH (1000 U, 213 U/mg solid) or L-LDH (1000 U, 813 U/mg solid) and FDH (120 U, 1.05 U/mg solid). The enzyme solution was transferred to a 10 cm length of dialysis tubing with a pore size to allow movement only of molecules smaller than 12–14 kDa across the membrane, and both ends were tied closed, excluding as much air as possible. The tubing was added to the flask and the pH was maintained by pH stat titration at 7.35 by the addition of 0.1 M HCl. After 11/10 days, when the theoretical volume of HCl had been added and the reaction rate approached zero, the enzymes in the dialysis tubing were removed from the flask, rinsed, and stored in 5 mM tris-HCl buffer in H_2_O at pH 7.5 at 5 °C. The reaction mixture was concentrated to 150 mL and acidified to pH 0.7 with concentrated H_2_SO_4_. Water (3 mL) was placed into the receiving flask of a continuous solvent extractor and the concentrated, acidified reaction mixture was continuously extracted into diethyl ether for 3 days. Evaporation of the diethyl ether in vacuo at 25 °C provided D-lactic acid-*d*_4_ as an 85% solution in H_2_O (8.53 g, 87%, 97% D) and L-lactic acid-*d*_4_ as an 85% solution in H_2_O (8.32 g, 85%, 95% D).

### 3.3. (L,L)-Lactide-d_8_ (***4***) and (D,D)-Lactide-d_8_ (***5***)

Lactides were synthesized from the lactic acids in the presence of zeolite Beta as a catalyst. In a typical example, 7.30 g of L-lactic acid-*d*_4_ (85% solution) was mixed with 60 mL of toluene and 4.26 g of zeolite Beta (Si/Al = 12.5). The mixture was heated overnight using a Dean-Stark apparatus to remove water. The Dean-Stark apparatus was then replaced by a reflux condenser, which allowed for the extraction of residual water in the back-flowing toluene with the help of 35 g of 4 Å molecular sieves (see SI, Figure S13). This process was continued for 7 h. The hot toluene solution was filtered and the solvent was removed under reduced pressure. After vacuum drying for 4 h, 3.95 g of raw product was obtained. In the purification step, the raw product was first sublimed at a pressure of about 10^−3^ mbar and 70 °C using a standard sublimation apparatus. Finally, it was recrystallized inside a glove box from 12 mL of dry toluene at 3 °C, centrifuged and washed with 3 mL of dry toluene. After vacuum drying for 3 h, 2.02 g of (L,L)-lactide-*d*_8_ (**4**) was obtained. In order to optimize the yield, the zeolite material used for the lactide formation was heated with 30 mL of water to 70 °C. After one week, the zeolite was removed by filtration and the solid residues from the sublimation and recrystallisation processes were added to the aqueous solution. The mixture was again heated to 70 °C for one week. Toluene (25 mL) was added and most of the water was removed using a Dean-Stark apparatus. After the addition of another 45 mL of toluene and 1.2 g of zeolite Beta, the process described above was repeated, yielding 2.08 g of raw product after the lactide formation and 1.10 g of purified product. Using the same procedure as described for (L,L)-lactide-*d*_8_, the treatment of 8.10 g of D-lactic acid-*d*_4_ (85% solution) resulted in 3.55 g of (D,D)-lactide-*d*_8_ (**5**).

### 3.4. Perdeuterated Poly-D-Lactic Acid (***6***) and Perdeuterated Poly-L-Lactic Acid (***7***)

All operations were performed in a glovebox under an argon atmosphere except when heating was required, in which case the experiments were started in Schlenk flasks in a glovebox and the flask was connected to a Schlenk line immediately afterwards. Vials and flasks were dried in an oven at 130 °C and introduced into the glovebox while still warm.

#### Polymerization Procedure

A stock solution of 137 mg diethylzinc (1.1 mmol) in 10 mL THF (conc. 0.11 M) and a stock solution of 108 mg benzyl alcohol (1.0 mmol) in 5 mL THF (conc. 0.2 M) were prepared. Aliquots (250 µL) of each stock solution were transferred to a small vial, mixed and left to react for 30 min, resulting in pale yellow initiator solutions. During this pre-reaction time, the given amounts of (D,D)-lactide-*d*_8_ were dissolved in 2.5 mL THF in vials or Schlenk flasks equipped with magnetic stirring bars. To start the reaction, 100 µL of the initiator solution were transferred to the vials or Schlenk flasks using a 200 µL glass syringe. Aliquots of about 50 mg were transferred to NMR tubes after the given times, and the reaction was quenched by addition of 0.5 mL of wet chloroform-*d*_1_ (CDCl_3_). These NMR samples were subsequently dried under vacuum and the resulting solids were taken up in THF for SEC analysis. The bulk reactions were quenched by the addition of a few drops of methanol in air. Polymers were isolated by injection of the reaction mixtures into a 10-fold excess of pentane containing 10% of 2-propanol. These mixtures were left to settle in the fridge overnight and filtered. Solids were dried to constant weight in a vacuum oven at 40 °C.

The polymerization experiments on the gram scale were carried out in the same way. Perdeuterated D-PLA was synthesized using 0.872 g of (D,D)-lactide-*d*_8_ dissolved in 6 mL of dry THF, 0.137 mmol of benzyl alcohol, and 0.152 mmol of diethylzinc. The reaction was quenched after 24 h, and after polymer isolation, 0.848 g (96%) of perdeuterated D-PLA (**6**) was obtained. For perdeuterated L-PLA, 1.059 g of (L,L)-lactide-*d*_8_ was dissolved in 7 mL of dry THF; 0.168 mmol of benzyl alcohol and 0.194 mmol of diethylzinc were used and 1.03 g (96%) of perdeuterated L-PLA (**7**) was obtained. SEC analysis gave M_n_ (perdeuterated D-PLA **6**) = 6850 g/mol, M_w_/M_n_ (perdeuterated D-PLA **6**) = 1.09 (theoretical M_n_ at full monomer conversion: 6470 g/mol) and M_n_ (perdeuterated L-PLA **7**) = 6330 g/mol, M_w_/M_n_ (perdeuterated L-PLA **7**) = 1.17 (theoretical M_n_ at full monomer conversion: 6410 g/mol).

## 4. Results and Discussion

**Scheme 1:**
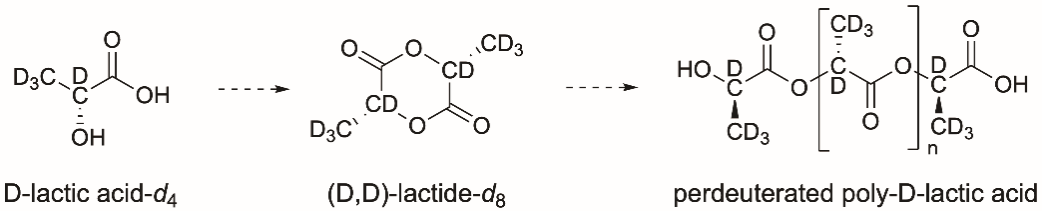
Synthetic strategy to stereodefined, perdeuterated polylactic acid via conversion of enantiopure lactic acid-*d*_4_ monomers to enantiopure lactide-*d*_8_ dimers.

Our synthetic strategy was to produce perdeuterated enantiopure samples of D- and L-lactic acid-*d*_4_, from which we could synthesize the perdeuterated polymers via the enantiopure lactides (Scheme 1, D-enantiomer).

Deuterium-labeling is necessary to investigate the PLA polymers using neutron scattering, NMR spectroscopy, and Raman microspectroscopy tools. The monomers, dimers, and polymers are not suitable substrates for direct H/D exchange. Furthermore, lactic acid polymers have many applications for which the material performance depends on the details of the microstructure [1] and it was therefore necessary to develop a synthetic route to perdeuterated PLA polymers with well-defined chirality.

**Scheme 2:**
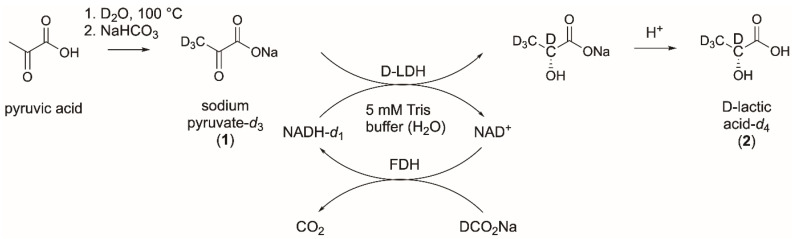
Enzymatic synthesis of perdeuterated D-lactic acid-*d*_4_ (**2**) from sodium pyruvate-*d*_3_ (**1**) using D-lactate dehydrogenase (D-LDH) enzymes, with concomitant generation of the deuterated co-enzyme NADH-*d*_1_ via formate dehydrogenase-catalyzed oxidation of sodium formate-*d*_1_.

We began with the prochiral precursor pyruvate, which is easily perdeuterated in D_2_O [6], and used immobilized enzymes as the catalysts for synthesizing enantiopure lactic acid-*d*_4_ monomers from this common precursor. We could select the chirality of the perdeuterated lactic acid monomers by selecting either D-LDH or L-LDH enzymes. The deuterium source for the enantiospecific reduction of pyruvate is commercially available sodium formate-*d*_1_ (Scheme 2) [19]. A key advantage of the method is that the solvent is not involved in the deuterium transfer processes [20] and the reaction therefore can be carried out in (Tris-buffered) H_2_O [19].

The conditions for the enzymatic reaction depicted in Scheme 2 were established with an unlabeled system to produce unlabeled D-lactic acid using D-LDH. The reaction was performed using membrane-enclosed enzymatic catalysis (MEEC) [15] in a dialysis bag, for the operational simplicity of this system, and to facilitate the reuse of the enzymes. The reaction took several days to reach completion, determined by pH-stat titration with 0.1 M HCl. Argon was bubbled constantly through the reaction chamber to reduce inactivation of the enzymes by oxidation and to remove the CO_2_ produced by formate oxidation. The polymerization to polylactic acid requires monomers of very high purity, and for this reason, we omitted the use of reducing agents such as dithiothreitol or β-mercaptoethanol which are often used to minimize oxidation of enzymes [12,14]. Concentration, acidification, and continuous extraction of the reaction mixture into diethyl ether over three days, followed by removal of the diethyl ether under reduced pressure, provided D-lactic acid (**8**) as an 85% solution in H_2_O.

The synthesis of perdeuterated D-lactic acid-*d*_4_ (**2**) was initially performed on a 4.4 mmol scale using the same D-LDH/FDH solution in four subsequent cycles. No significant loss of enzyme activity was observed (see SI Figure S9). The system was amenable to scale-up and was subsequently performed on an 89 mmol scale, allowing isolation of a multi-gram quantity of D-lactic acid-*d*_4_ (**2**) as a concentrated solution in water. The deuterium incorporation could be assessed qualitatively by ^13^C NMR (the splitting patterns of the methine and methyl carbon signals were consistent with high level of deuterium incorporation, see SI Figure S4) and quantitatively by high resolution mass spectrometry, comparing the intensities of the isotopologues (see SI Figure S5). The non-exchangeable protons of D-lactic acid-*d*_4_ (**2**) were calculated to be >97% ^2^H by HRMS. The enantiomeric purity was determined by GC analysis of the menthyl ester derivative (**9**), with none of the other diastereoisomer detected (see SI Figure S11).

The procedure was repeated using L-LDH to synthesize L-lactic acid-*d*_4_ (**3**). Enzyme activity was assessed on a small scale, and during the fourth cycle, some loss of activity was observed (see SI Figure S10). The method was also used on a multi-gram scale to prepare a large quantity of L-lactic acid-*d*_4_ (**3**). The enantiomeric purity was confirmed by GC analysis of the menthyl ester derivative (**10**), with none of the other diastereoisomer detected (see SI Figure S12). The deuterium incorporation was assessed using the same methods as for the D-enantiomer and calculated to be 94% ^2^H by HRMS (see SI Figure S8).

The specific optical rotation values of D-lactic acid-*d*_4_ (**2**; +12.1°) and L-lactic acid-*d*_4_ (**3**; −12.3°) suggest high enantiopurity.

### 4.1. Lactide Synthesis and Characterization

First, test experiments were executed using commercial, unlabeled L-lactic acid (>95% L-form) and zeolite Beta catalysts in their protonated form with Si/Al ratios of 12.5 or 15. The relatively high amount of Al is necessary to accelerate the acid-catalyzed condensation reaction. Prior to lactide formation, the lactic acid samples (70–90% in water) were diluted to 50% with extra water and stirred under argon at 70 °C for one week. During this time, co-existing oligomeric linear polylactic acid chains were hydrolyzed, and the reaction was monitored by ^1^H NMR.

**Figure 1:**
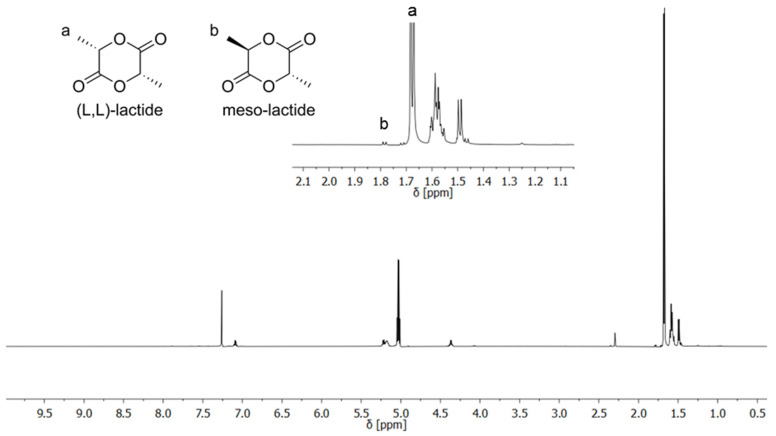
^1^H NMR spectrum in CDCl_3_ of crude (L,L)-lactide (**11**). The methyl groups of (L,L)-lactide and meso-lactide and their corresponding signals are denoted a and b respectively.

The reaction conditions were optimized in terms of temperature, catalyst, and reaction time. Under the optimized conditions, zeolite Beta (Si/Al = 12.5) was suspended in toluene and lactic acid was added. The mixture was stirred under reflux and water was trapped in a Dean-Stark apparatus. The solution was filtered, and the solvent evaporated. Figure 1 shows the ^1^H NMR spectrum of crude (L,L)-lactide (**11**). The major, desired product, (L,L)-lactide (**11**), is represented by the dominant doublet at 1.68 ppm (*J* = 4.4 Hz) and quartet at 5.03 ppm (*J* = 4.4 Hz). The signal at 1.78 ppm is attributed to the methyl groups of meso-lactide, which is present in trace quantities. The signals at 4.37 ppm (quartet, *J* = 4.6 Hz) and 5.22 ppm (quartet, *J* = 4.7 Hz) represent the methine protons of the open dimer and higher oligomers, as well as unreacted lactic acid, present in total in quantities of about 10%. Signals at 1.49 ppm (doublet, *J* = 4.6 Hz) and 1.55–1.61 ppm (complex) arise from the methyl groups of these impurities.

The polymerization degrees of the higher oligomers could be determined by additional HPLC-MS/UV measurements to be between 3 and 6. The high enantiopurity of the lactide was confirmed by GC-FID (see SI, Figure S14). Only small traces of meso-lactide were found in the crude product, most likely arising from impurities in the commercial starting material. In addition, the overall yield of the lactide formation step could be increased to more than 60%, although the sample quantities were only in the gram scale.

**Figure 2:**
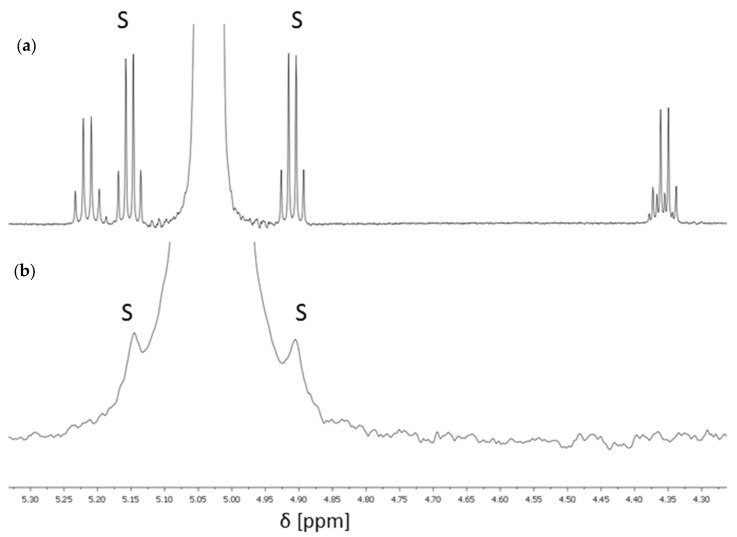
Sections of (**a**) the ^1^H NMR spectrum of unlabeled (L,L)-lactide (**11**) (in CDCl_3_) and (**b**) the ^2^H NMR spectrum of perdeuterated (D,D)-lactide-*d*_8_ (in CDCl_3_) comparing the methine signals. The peaks indicated with S represent the ^13^C satellites of the lactide signals.

In order to remove lactic acid and linear polylactic acid oligomers, the crude products were purified by sublimation and subsequent recrystallisation from toluene. Nevertheless, the products still contained approximately 1% of the open dimer. For this reason, the water removal process during the lactide formation was optimized for the syntheses of the perdeuterated lactides. After removal of most of the water, the Dean-Stark apparatus was replaced by a modified reflux condenser, in which the solvent back-flow was dried using molecular sieves (see SI, Figure S13). The water content of toluene is 0.03% at 25 °C, which was approximately the temperature in the separation zone of the Dean-Stark apparatus. With the help of a molecular sieve, the water content could be reduced by one order of magnitude. As the lactide formation is an equilibrium reaction, the reduced water concentration reduces the amount of non-lactide products. In addition, the final recrystallisation step was carried out using dry toluene inside a glove box in order to suppress a possible back reaction of already formed lactide to the open dimer. As a result, no other products than the lactide could be detected by NMR spectroscopy. Figure 2a shows the methine proton region of the ^1^H NMR spectrum of purified unlabeled (L,L)-lactide (**11**)**.** In this case, the additional water removal with molecular sieves was not applied and the recrystallization step was carried out using non-dry toluene. As a consequence, the product contains 1% of open dimer (signals at 4.37 and 5.22 ppm) and about 0.3% of lactic acid (additional smaller quartet at 4.37 ppm). Figure 2b shows the methine proton region of the ^2^H NMR spectrum of purified perdeuterated (D-D)-lactide-*d*_8_ (**5**), which was synthesized with the help of molecular sieves and recrystallized using dry toluene. In this case, no open dimer or lactic acid was detectable. The signal width and the signal to noise ratio of ^2^H NMR spectra are less favorable than in the case of ^1^H NMR spectra. Nevertheless, the satellites of the lactide signal at 5.0 ppm are clearly visible. Each of them represents 0.55% of the intensity of the main signal. This indicates that the impurity level of the perdeuterated lactide must be well below this value.

The specific optical rotation of (D,D)-lactide-*d*_8_ (**5**; +257.5°) was determined in dichloromethane (DCM) and is in agreement with the reported value for unlabeled (L,L)-lactide in DCM (−266.3°) [31].

### 4.2. Polymerization

**Figure 3:**
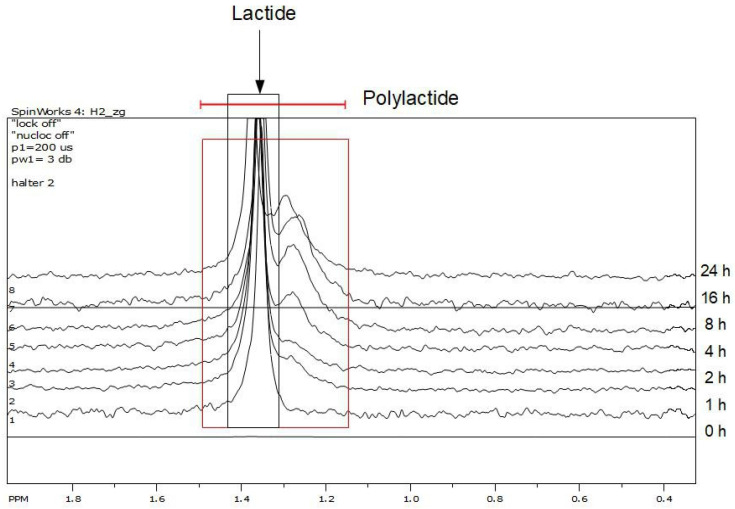
Methyl region of the ^2^H NMR spectra of the samples from run 1a–f (Table 1) showing the overlapping signals of the (D,D)-lactide-*d*_8_ (**5**) monomer (1.30 to 1.45 ppm) and the perdeuterated poly-D-lactic acid (**6**) polymer (1.15 to 1.50 ppm).

To get an overview of the polymerization behavior of the perdeuterated (D,D)-lactide-*d*_8_ (**5**), six individual runs were performed using diethylzinc/benzyl alcohol as the initiator system (Table 1). At ambient temperature (a.t.), two runs were performed at a target monomer to initiator ratio of 100:1. Two more runs were carried out at ambient temperature, using target monomer to initiator ratios of 50:1 and 200:1, respectively. An additional two runs at 50 °C were performed in a similar way to confirm the results and to monitor the reaction. Conversions were estimated by integration of the 2D NMR spectra, as the signals of the monomer and polymer overlap (Figure 3). The spectra indicate that the conversion comes to an end at about 50 to 60% of monomer consumption under the chosen conditions.

While the runs with around 100 equivalents of monomer show a reasonable agreement between conversions determined by NMR spectroscopy and by isolation, isolated yields are quite different from NMR spectroscopy results for the 50 and 200 monomer equivalent experiments due to the small sample size leading to significant losses during filtration (for 50 equiv.) and insufficient separation (for 200 equiv.). The apparent loss of polymerization activity at around 50% to 60% of deuterated monomer conversion is different from unlabeled systems where usually more than 90% of monomer conversion was obtained under the same polymerization conditions. The incomplete conversion of the perdeuterated monomers indicates the presence of deactivation reactions of the zinc-organic head groups.

**Figure 4:**
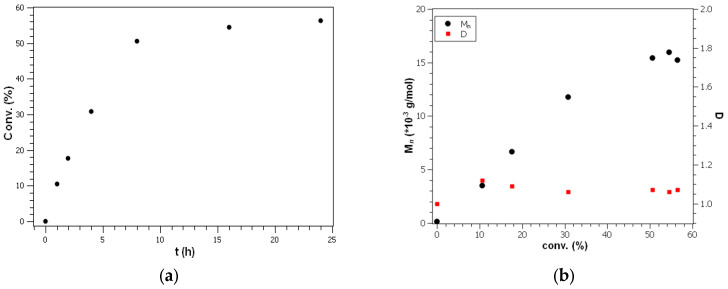
(**a**) Plot of (D,D)-lactide-*d*_8_ (**5**) conversion versus time; and (**b**) molecular weight (M_n_) and molecular weight distribution (D) versus (D,D)-lactide-*d*_8_ (**5**) conversion to perdeuterated D-PLA (**6**) (entries 1a–f, Table 1).

Figure 4 shows plots of conversion versus time as well as molecular weight versus conversion (entries 1a–f in Table 1) which demonstrate that the polymerization is well-controlled in the early stages. This is also reflected by the narrow molecular weight distributions D = M_w_/M_n_. After 24 h at ambient temperature (entry 1f), the molecular weight seems to decrease slightly, possibly due to the transesterification processes, although this is not yet reflected in the molecular weight distribution.

At 50 °C, the reaction started well-controlled (entry 5a) and at a much higher reaction rate, but transesterification became more prominent and led to broader molecular weight distributions (entries 5b and 6). Interestingly, the monomer conversion in these experiments was 85% and the final molecular weights were visibly above the ones obtained at ambient temperature. These results can be explained by the presence of continuous head group deactivation reactions probably caused by oxygen and moisture being present in small quantities even under inert gas conditions. In a slower polymerization reaction, the deactivation is more prominent, and, as a result, the monomer conversion is smaller. As a consequence of the limited amounts of perdeuterated monomers, the initiator quantities used for each polymerization experiment were only 5 µmol, which makes the deactivation scenario described above reasonable.

Additional polymerization experiments on the gram scale supported the above explanation. For both perdeuterated D-PLA (**6**) and perdeuterated L-PLA (**7**), the monomer conversion was 96%. In addition, the molecular weight distributions were narrow, and the measured molecular weights coincided well with the ones calculated from the quantities of initiator and monomer at full monomer conversion (Section 2).

**Figure 5:**
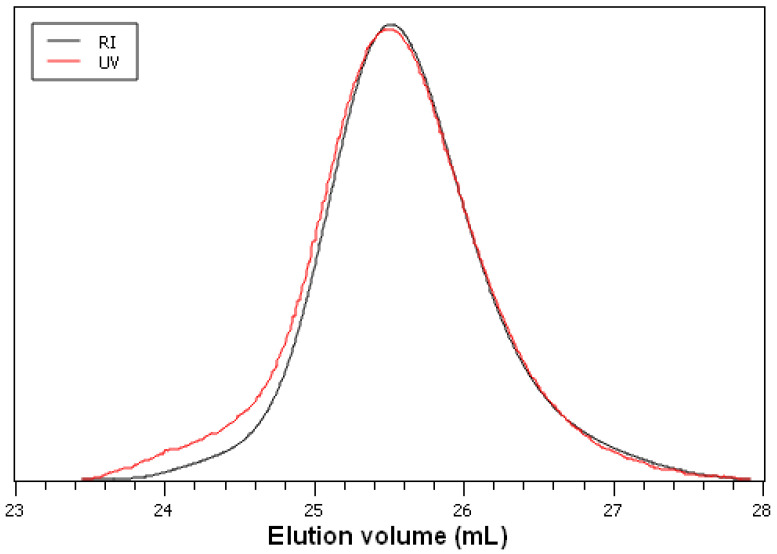
RI and UV SEC traces of the perdeuterated D-PLA (**6**) sample from entry 3 (Table 1).

Figure 5 shows the refractive index (RI) and UV SEC traces of the sample from entry 3 (Table 1). Both traces display a symmetric shape and underline the narrow molecular weight distribution of the perdeuterated poly-D-lactic acid (**6**). In summary, the polymerization behavior of the perdeuterated lactides is similar to that of unlabeled monomers observed before for the polymerization system chosen. Due to the high sensitivity of anionic polymerization reactions to impurities, the results underline the high level of purity of the perdeuterated precursors.

The polymers were additionally examined by NMR spectroscopy. Figures S16 and S17 show the ^13^C and homonuclear decoupled ^1^H signals of the methine group (entry 6, Table 1). The main signals at 68.99 ppm and 5.17 ppm represent the iii tetrades [22]. Tetrades with other stereo-orientations are present only in very small quantities. The DSC measurement of the same sample (Figure S18) shows a strong melting peak at 170 °C. The glass transition between 50 and 75 °C is only weakly pronounced, which is expected for highly crystalline PLA. Both NMR and DSC measurements underline a high degree of stereoregularity in the polymer chains.

## 5. Conclusions

In this work, we have reported a synthetic route for the preparation of stereodefined, perdeuterated polylactic acid (PLA), beginning with the enzymatic synthesis of enantiopure lactic acid-*d*_4_ from prochiral sodium pyruvate-*d*_3_. The source of the lactate dehydrogenase enzymes (rabbit muscle or *Lactobacillus leichmanii*) determines the chirality of the monomers. By coupling the enzymatic reduction of pyruvate-*d*_3_ with in situ generation of NADH-*d*_1_, we achieved high enantioselectivity and complete deuterium incorporation without the need for deuterated solvents. This approach offers a clean and stereoselective path to perdeuterated lactic acid monomers of high purity. The monomers were subsequently converted to high purity lactide-*d*_8_ stereoisomers using a zeolite-catalyzed route and polymerized via anionic ring-opening using a ZnEt_2_/ROH system.

This method enables the synthesis of perdeuterated polylactic acid polymers that are highly suitable for the characterization of polymer structure and dynamics using neutron scattering, infrared and nuclear magnetic resonance spectroscopy methods that benefit from deuterium-labeling. These perdeuterated polymers have recently been used to study the incorporation of microplastics into microbial cells [30] and show promise for understanding the pathways of microbial processing of PLA-based plastics [30].

## Figures and Tables

**Table 1:** Summary of polymerization results for (D,D)-lactide-*d*_8_ (**5**).

Entry	Amount (D,D)-Lactide-*d*_8_ (mg)	Equiv. (D,D)-Lactide-*d*_8_	T (°C)	t (h)	Conv. (%) NMR	Yield (mg)	Conv. (%) Weight	M_n_ (g/mol)	D
1a	145	95	a.t.	1	28	n.d.	n.d.	3480	1.12
1b	145	95	a.t.	2	29	n.d.	n.d.	6680	1.09
1c	145	95	a.t.	4	44	n.d.	n.d.	11,790	1.06
1d	145	95	a.t.	8	45	n.d.	n.d.	15,430	1.07
1e	145	95	a.t.	16	54	n.d.	n.d.	15,980	1.06
1f	145	95	a.t.	24	52	55	55 *	15,230	1.07
2	72	47	a.t.	24	59	10	14	8030	1.11
3	144	95	a.t.	24	54	73	51	15,520	1.08
4	287	189	a.t.	24	48	221	77	29,300	1.04
5a	154	101	50	4	79	n.d.	n.d.	29,760	1.07
5b	154	101	50	8	85	120	78	23,970	1.21
6	154	101	50	8	85	115	74	28,630	1.23

* Adjusted to the reduced reaction size due to taking samples 1a–e; n.d. = not determined; a.t. = ambient temperature. The number averaged molecular weight (M_n_) and molecular weight distribution (D = M_w_/M_n_, where M_w_ is the weight averaged molecular weight) were determined using SEC.

## Data Availability

The original contributions presented in this study are included in the article. Further inquiries can be directed to the corresponding authors.

## Supplementary Materials

The following supporting information can be downloaded at: https://www.mdpi.com/article/10.3390/bioengineering12060575/s1, Figure S1. ^13^C NMR spectrum of sodium pyruvate-*d*_3_ (**1**). Figure S2. High resolution mass spectrum of sodium pyruvate-*d*_3_ (**1**). Figure S3. ^1^H NMR spectrum of D-lactic acid-*d*_4_ (**2**). Figure S4. ^13^C NMR spectrum of D-lactic acid-*d*_4_ (**2**). Figure S5. High resolution mass spectrum of D-lactic acid-*d*_4_ (**2**). Figure S6. ^1^H NMR spectrum of L-lactic acid-*d*_4_ (**3**). Figure S7. ^13^C NMR spectrum of L-lactic acid-*d*_4_ (**3**). Figure S8. High resolution mass spectrum of L-lactic acid-*d*_4_ (**3**). Figure S9. Stability of the D-LDH/FDH catalytic system over four cycles. Figure S10. Stability of the L-LDH/FDH D catalytic system over four cycles. Figure S11. Gas chromatogram-mass spectrum of D-lactic acid-*d*_4_ menthyl ester (**9**). Figure S12. Gas chromatogram-mass spectrum of L-lactic acid-*d*_4_ menthyl ester (**10**). Figure S13. Modified reflux condenser for removal of residual water using a molecular sieve. Figure S14. GC-FID trace of crude (L,L)-lactide (**11**). Figure S15. ^2^H NMR spectrum of (D,D)-lactide-*d*_8_ (**5**). Figure S16. Tacticity of perdeuterated poly-D-lactic acid (**6**) (Table 1, entry 6) determined by ^13C^{^1^H} NMR spectroscopy. Figure S17. Tacticity of perdeuterated poly-D-lactic acid (**6**) (Table 1, entry 6) determined by ^1H^{^1^H} NMR spectroscopy. Figure S18. Differential-scanning calorimetry of perdeuterated poly-L-lactic acid (**6**) (Table 1, entry 6).

## Abbreviations

The following abbreviations are used in this manuscript:

LDH	lactate dehydrogenase
NADH	nicotinamide adenine dinucleotide (reduced form)
NAD^+^	nicotinamide adenine dinucleotide (oxidized form)
FDH	formate dehydrogenase
PLA	polylactic acid
HRMS	high resolution mass spectrometry
QTOF	quadrupole time-of-flight
NMR	nuclear magnetic resonance
GC-MS	gas chromatography–mass spectrometry
SEC	size exclusion chromatography
DSC	differential scanning calorimetry
THF	tetrahydrofuran
CDCl_3_	chloroform-*d*_1_
MEEC	membrane-enclosed enzymatic catalysis
