# Supplementary material for: The Enzymatic Synthesis of Perdeuterated D- and L-Lactic Acid-d4 and Polymerization of Their Lactides to Polylactic Acid
Source: Bioengineering (Basel). 2025 May 27;12(6):575. doi: 10.3390/bioengineering12060575 (PMC12189507; doi:10.3390/bioengineering12060575)
Supplement: Supplementary file 1 [file bioengineering-12-00575-s001.zip › bioengineering-3591557-supplementary.pdf]

SUPPORTING INFORMATION

The enzymatic synthesis of perdeuterated D- and L-lactic acid-d_4 and polymerization of their lactides to polylactic acid.

Anna E. Leung[1]*, Andreas Raba[2], Klaus Beckerle[3], Jürgen Allgaier[2]*, Hanna P. Wacklin-Knecht[1,4]

[1]*European Spallation Source ERIC, PO Box 176, Lund, Sweden*
[2]*Jülich Centre for Neutron Science (JCNS-1), Forschungszentrum Jülich GmbH, Leo Brandt Straße, 52425 Jülich, Germany*
[3]*Institute for Inorganic Chemistry, RWTH Aachen University, Germany*
[4]*Division of Physical Chemistry, Department of Chemistry, Lund University, Sweden*

*Corresponding authors, email address: annaleung@live.com.au, j.allgaier@fz-juelich.de.

Figures:

S1. ^{13}C NMR spectrum of sodium pyruvate-d_3 (**1**)
S2. High resolution mass spectrum of sodium pyruvate-d_3 (**1**)
S3. ^{1}H NMR spectrum of D-lactic acid-d_4 (**2**)
S4. ^{13}C NMR spectrum of D-lactic acid-d_4 (**2**)
S5. High resolution mass spectrum of D-lactic acid-d_4 (**2**)
S6. ^{1}H NMR spectrum of L-lactic acid-d_4 (**3**)
S7. ^{13}C NMR spectrum of L-lactic acid-d_4 (**3**)
S8. High resolution mass spectrum of L-lactic acid-d_4 (**3**)
S9. Stability of the D-LDH/FDH catalytic system over four cycles
S10. Stability of the L-LDH/FDH D catalytic system over four cycles
S11. Gas chromatogram-mass spectrum of D-lactic acid-d_4 menthyl ester (**9**)
S12. Gas chromatogram-mass spectrum of L-lactic acid-d_4 menthyl ester (**10**)
S13. Modified reflux condenser for removal of residual water using a molecular sieve
S14. GC-FID trace of crude (L,L)-lactide (**11**)
S15. ^{2}H NMR spectrum of (D,D)-lactide-d_8 (**5**)
S16. Tacticity of perdeuterated poly-D-lactic acid (**6**) (Table 1, entry 6) determined by ^{13C}{^{1}H} NMR spectroscopy
S17. Tacticity of perdeuterated poly-D-lactic acid (**6**) (Table 1, entry 6) determined by ^{1H}{^{1}H} NMR spectroscopy
S18. Differential-scanning calorimetry of perdeuterated poly-D-lactic acid (**6**) (Table 1, entry 6)

Other:

Characterisation data for compounds **1-3**; **5**.

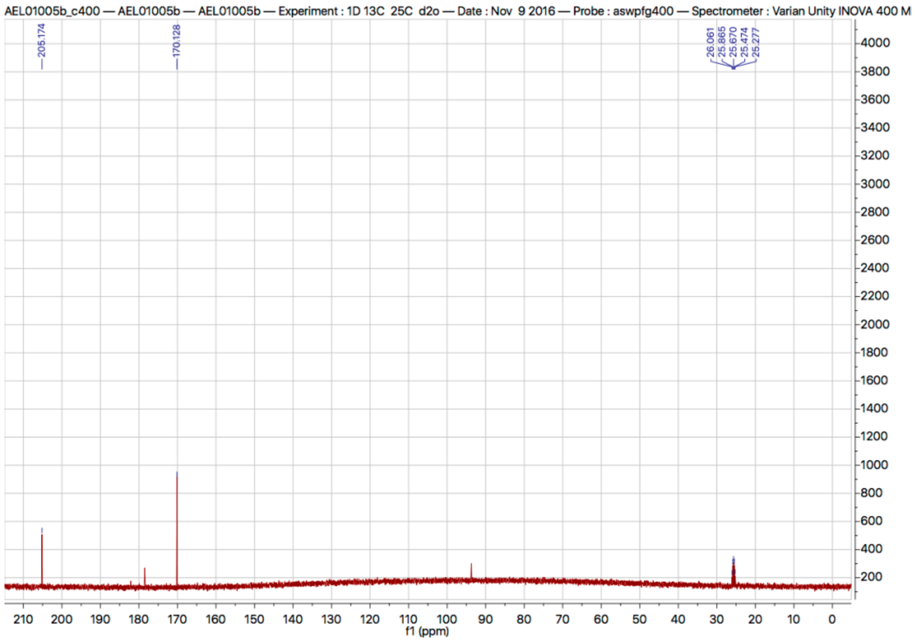

Figure S1. ^{13}C NMR spectrum of sodium pyruvate-d_3(**1**) (100 MHz, D_2O).

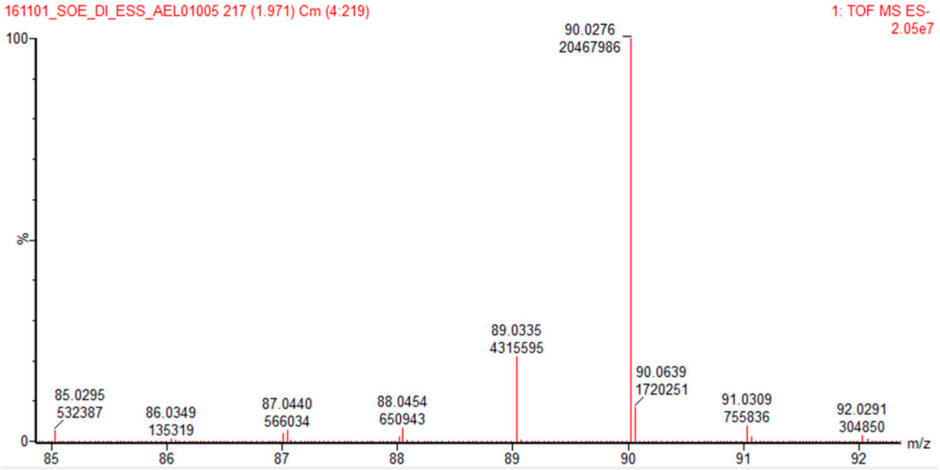

Figure S2. High resolution mass spectrum of sodium pyruvate-d_3 (**1**).

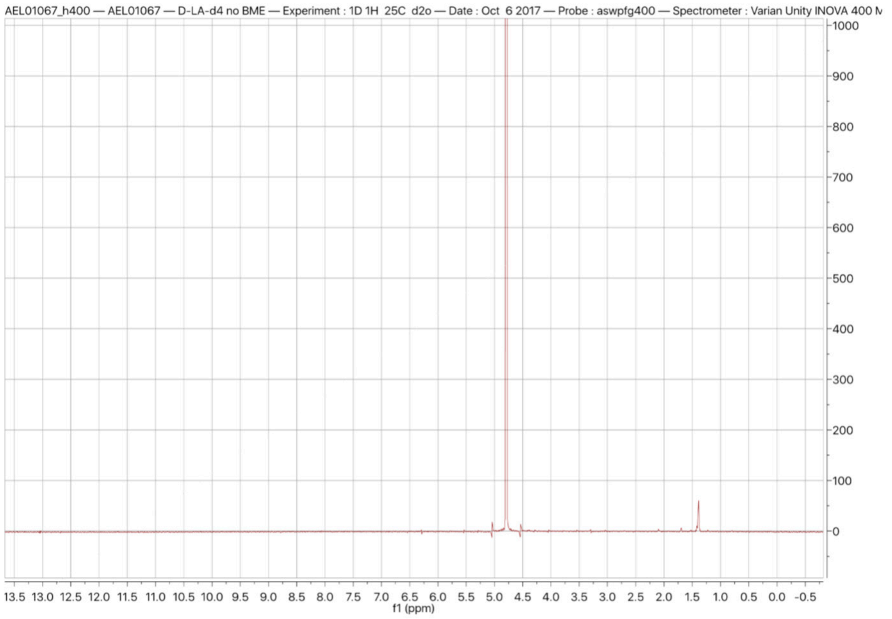

Figure S3. ^{1}H NMR spectrum of D-lactic acid-d_4 (**2**) (400 MHz, D_2O). Water and residual protons from the methyl group of D-lactic acid-d_4 are visible. Referenced to residual solvent.

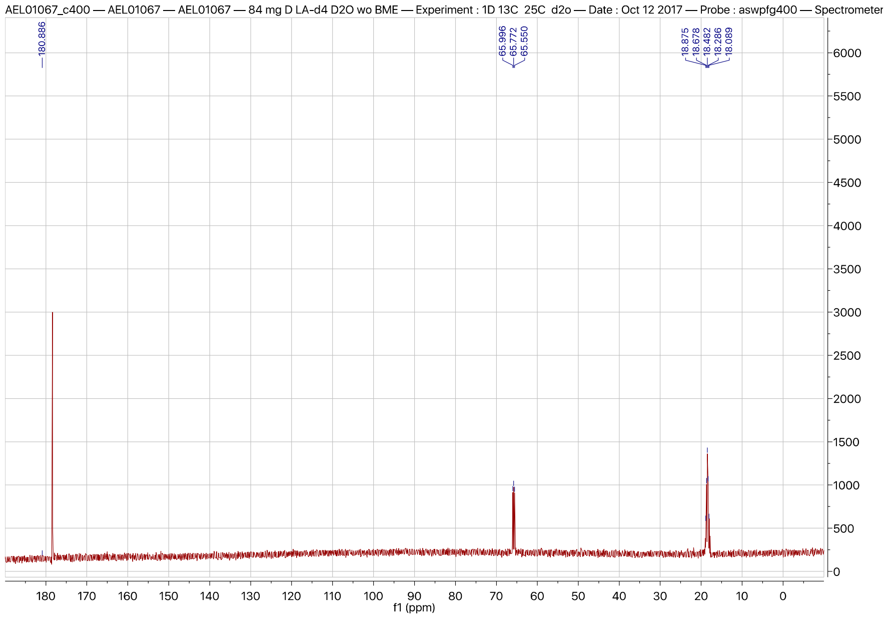

Figure S4. ^{13}C NMR spectrum of D-lactic acid-d_4 (**2**) (100 MHz, D_2O).

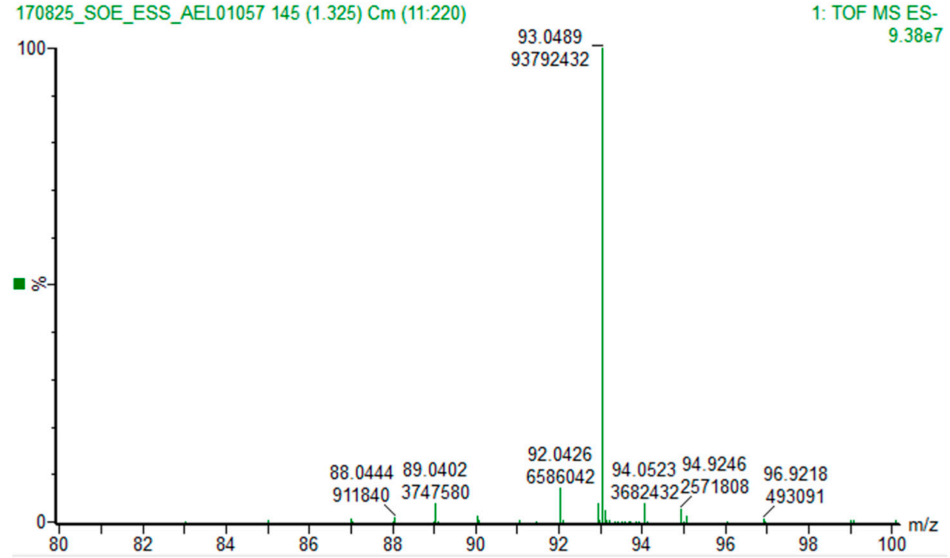

Figure S5. High resolution mass spectrum of D-lactic acid-d_4 (**2**).

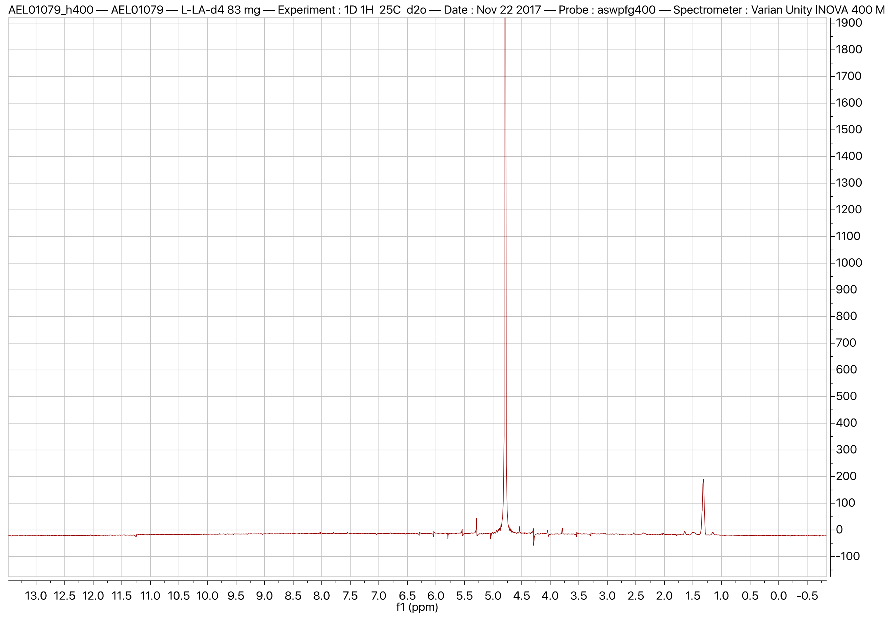

Figure S6. ^{1}H NMR spectrum of L-lactic acid-d_4 (**3**) (400 MHz, D_2O). Water and residual protons from the methyl group of L-lactic acid-d_4 are visible. Referenced to residual solvent.

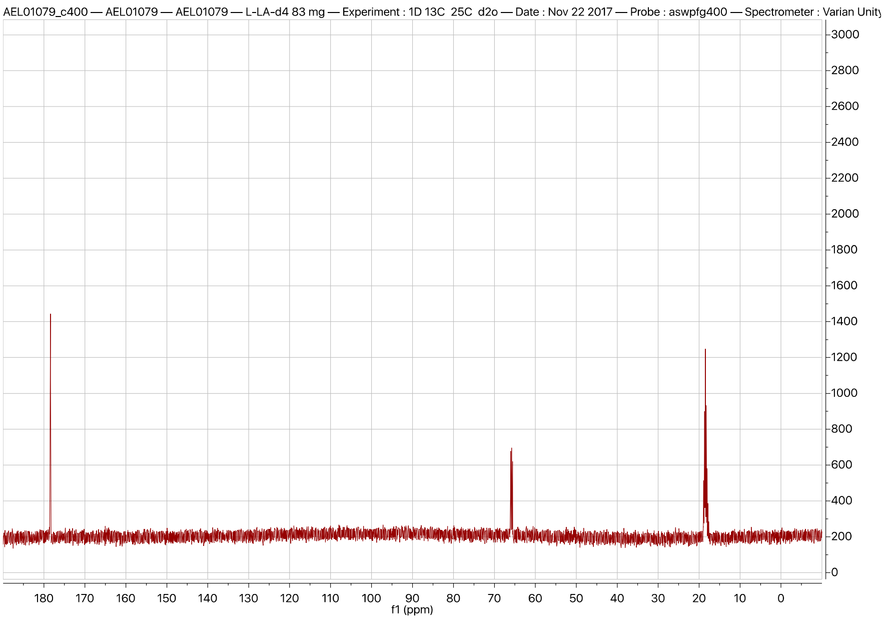

Figure S7. ^{13}C NMR spectrum of L-lactic acid-d_4 (**3**) (100 MHz, D_2O).

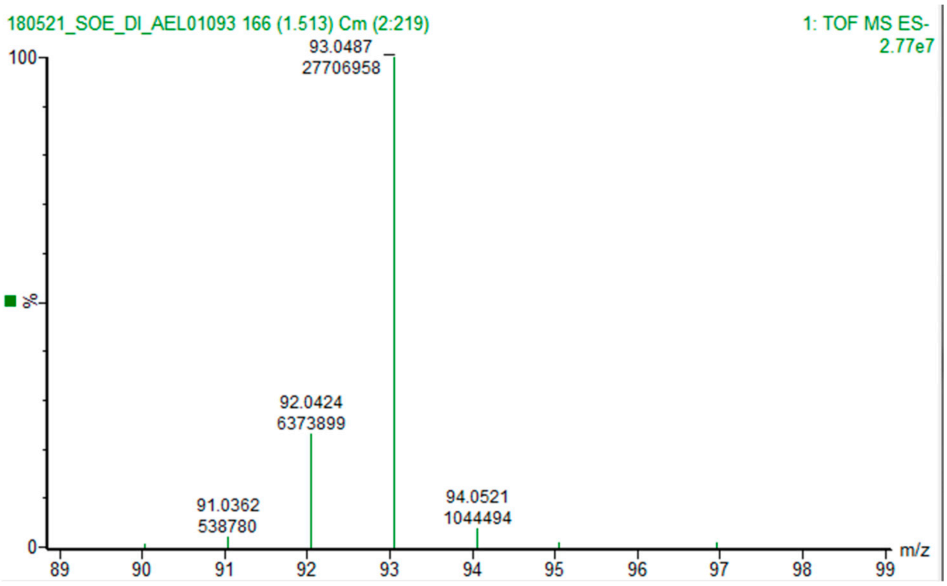

Figure S8. High resolution mass spectrum of L-lactic acid-d_4 (**3**).

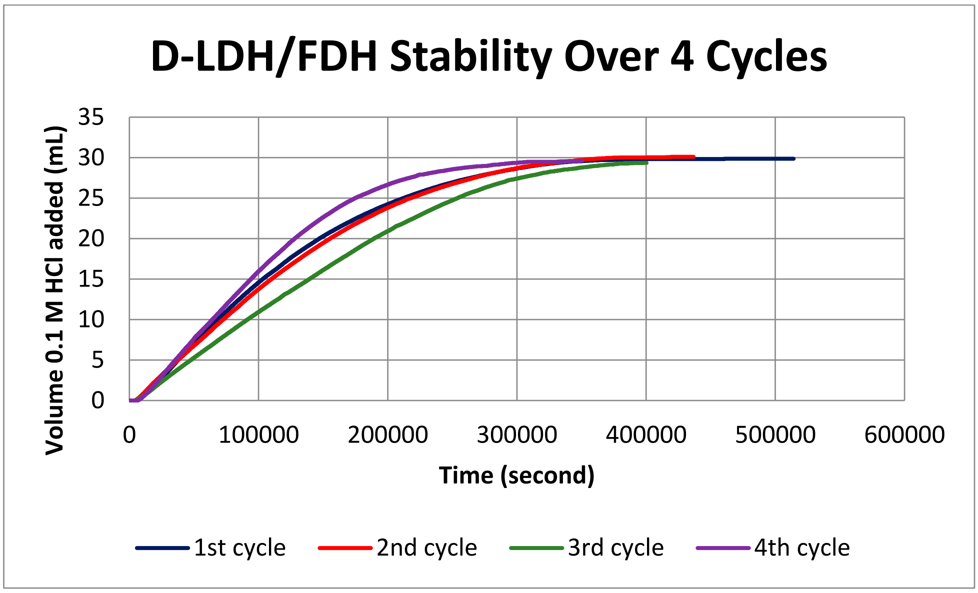

Figure S9. D-Lactate dehydrogenase/formate dehydrogenase activity over four cycles (assessed by the amount of 0.1 M HCl added to maintain a constant pH). The D-LDH/FDH system showed no significant loss of activity even during the fourth cycle.

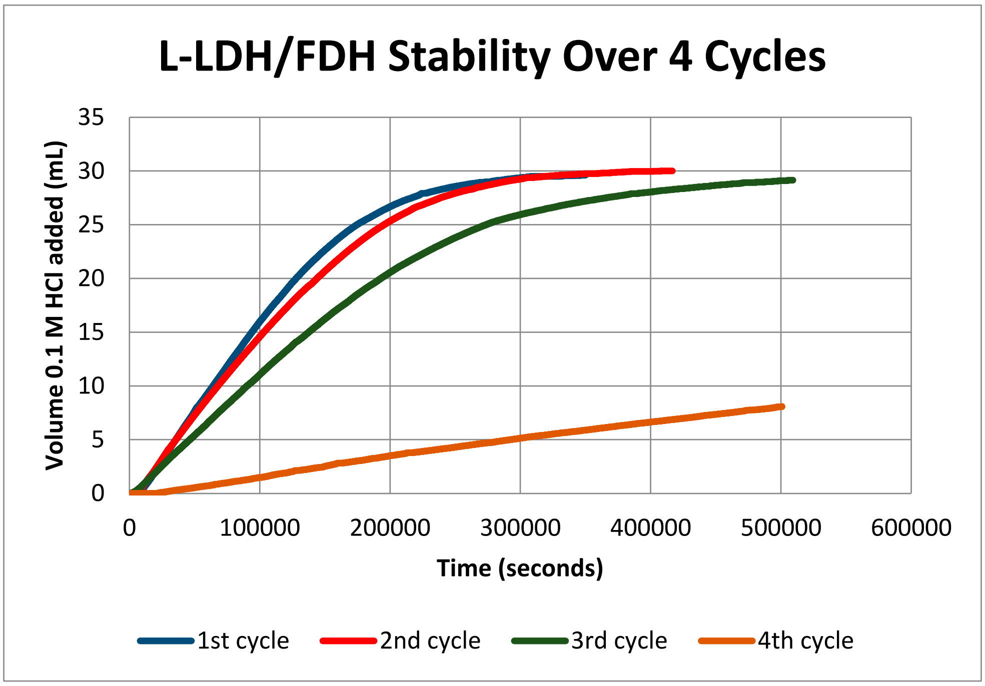

Figure S10. L-Lactate dehydrogenase/formate dehydrogenase activity over four cycles (assessed by the amount of 0.1 M HCl added to maintain a constant pH). The activity of the L-LDH/FDH system decreased during the third cycle, and by the fourth was decreased so much as to no longer be useful.

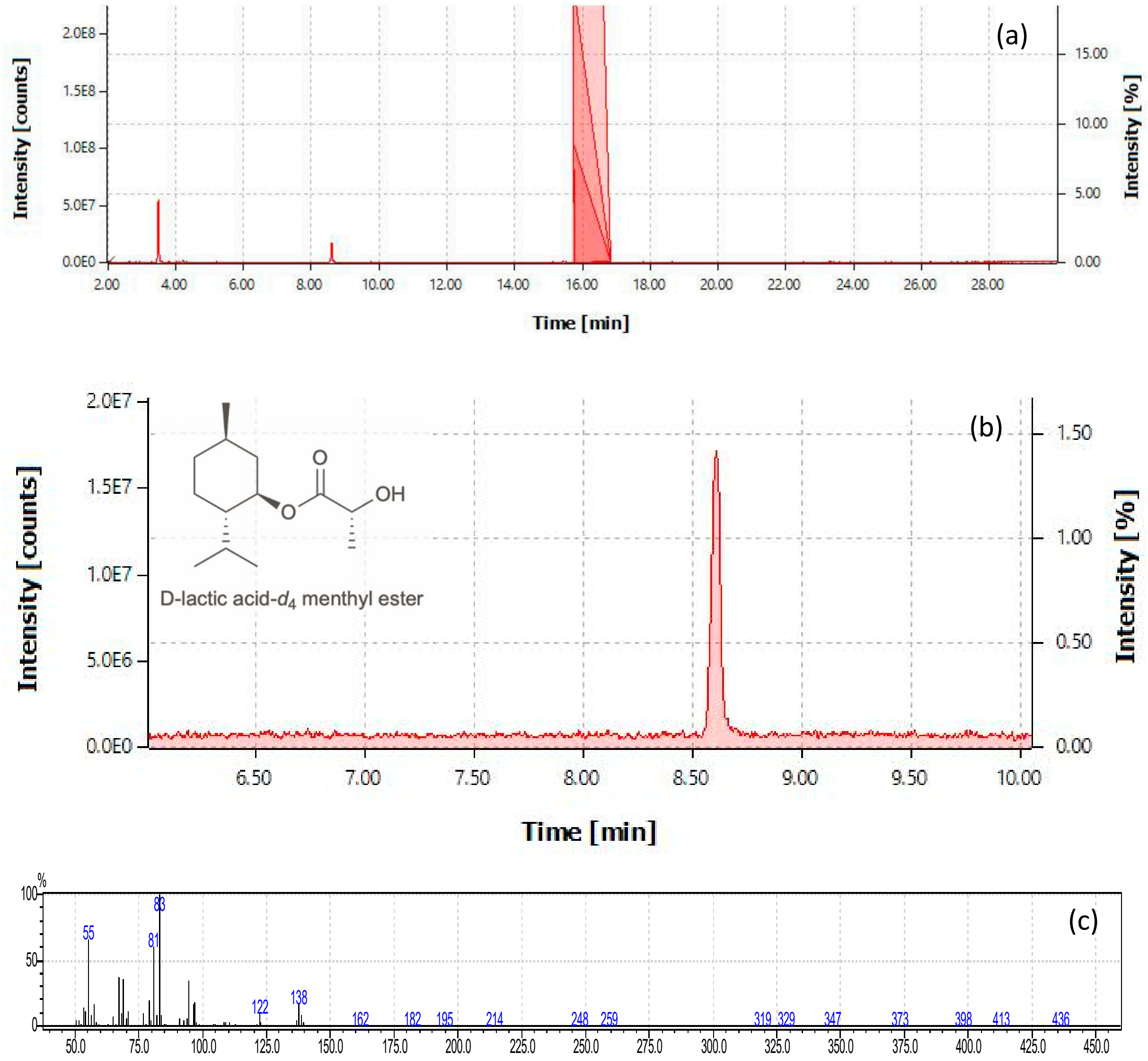

Figure S11. Gas chromatogram (a, b) and mass spectrum (c) for D-lactic acid-d_4 menthyl ester (**9**).

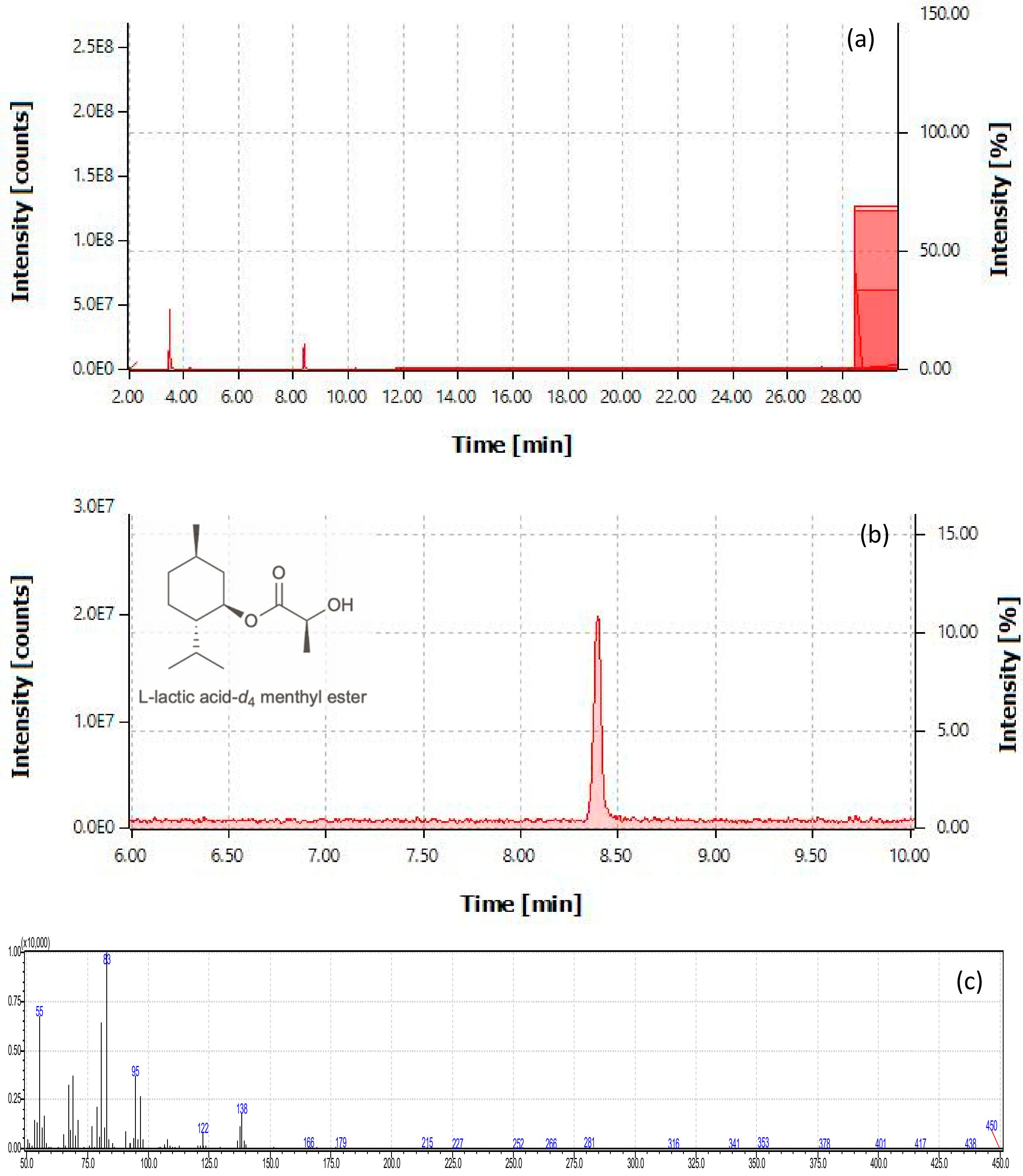

Figure S12. Gas chromatogram (a, b) and mass spectrum (c) for L-lactic acid-d_4 menthyl ester (**10**).

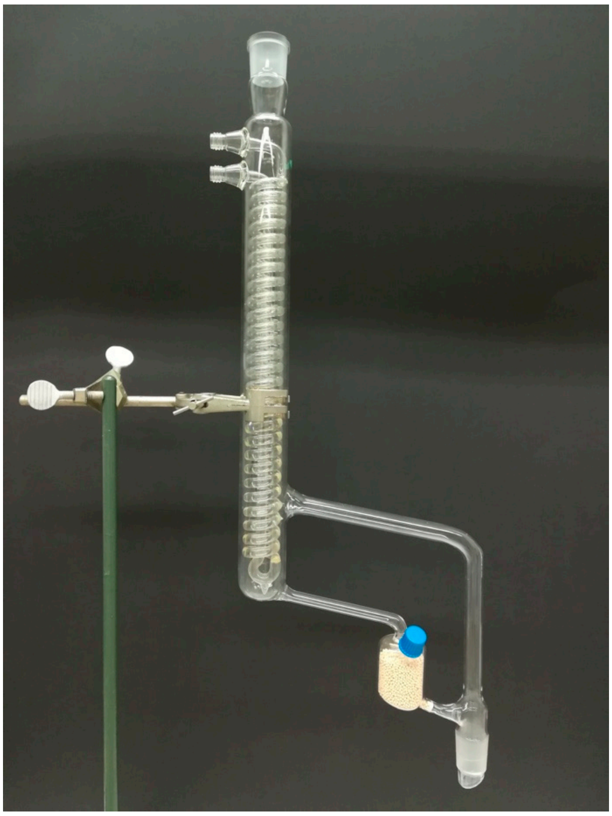

Figure S13. Modified reflux condenser containing a zone, where the back flowing solvent was dried using a molecular sieve during lactide synthesis.

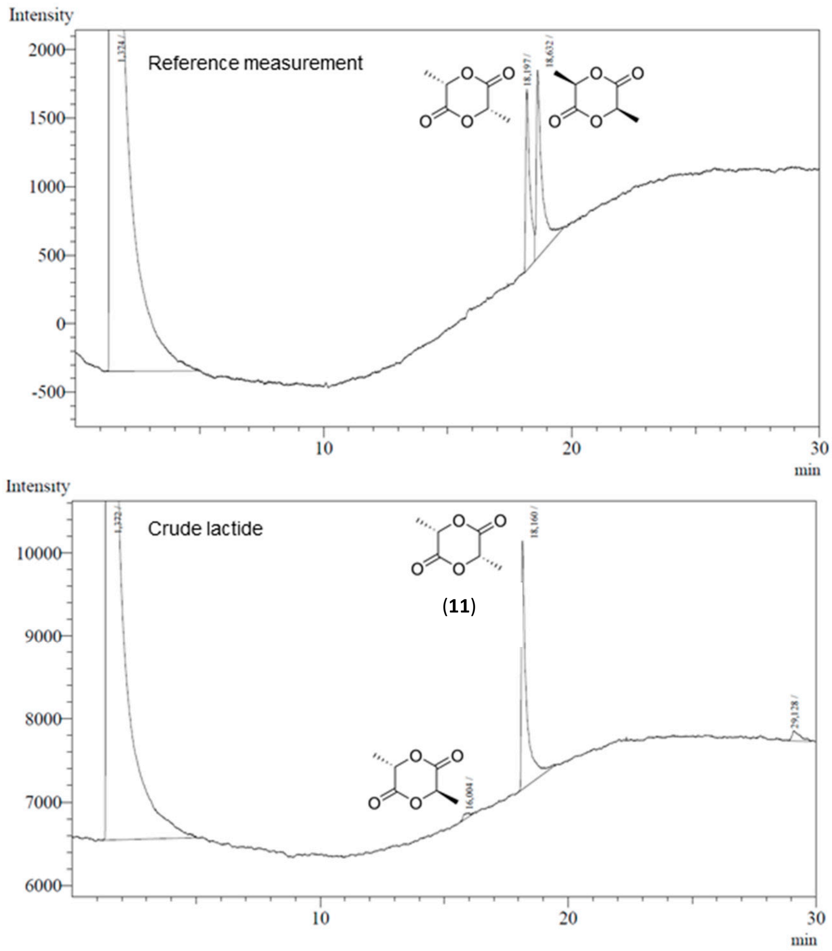

Figure S14. GC-FID trace of crude (L-L)-lactide (**11**) using a chiral column for separation: upper part: reference measurement of *rac*-lactide; lower part: crude (L-L)-lactide (**11**) with traces of *meso*-lactide

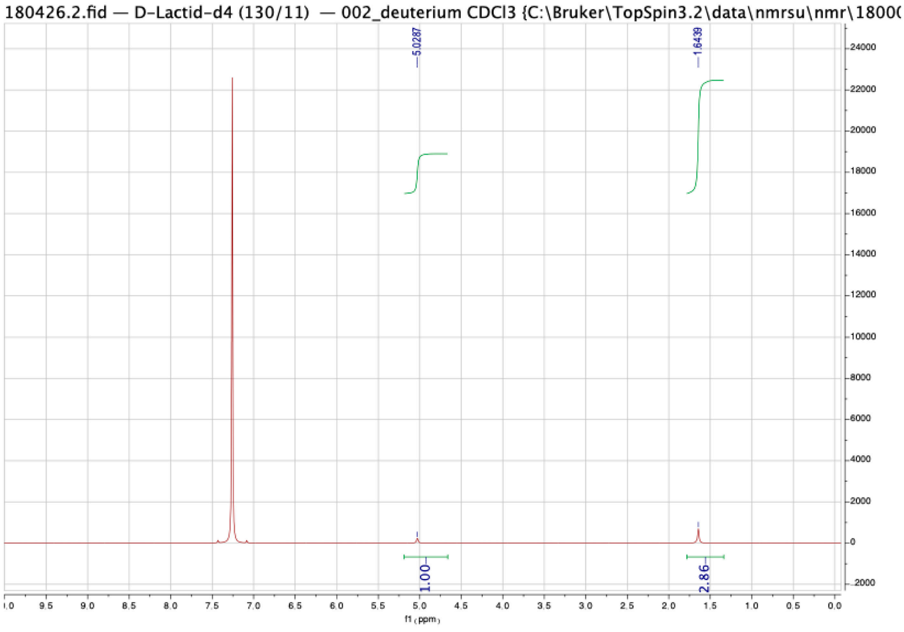

Figure S15. ^{2}H NMR spectrum of (D,D)-lactide-d_8 (**5**) (61 MHz, $CDCl_3$).

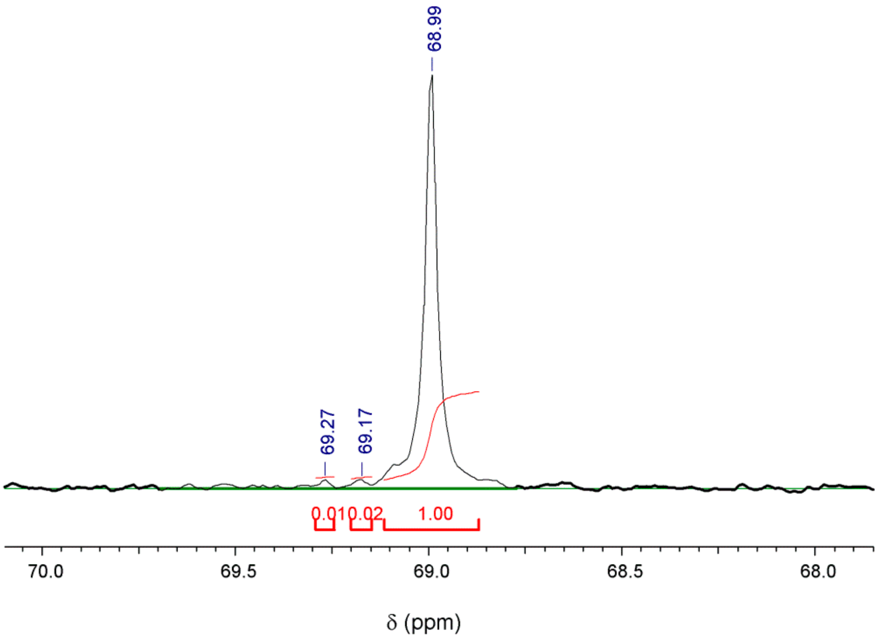

Figure S16. Tacticity of perdeuterated poly-D-lactic acid (**6**) (Table 1, entry 6) determined by ^{13C}{^{1}H} NMR spectroscopy.

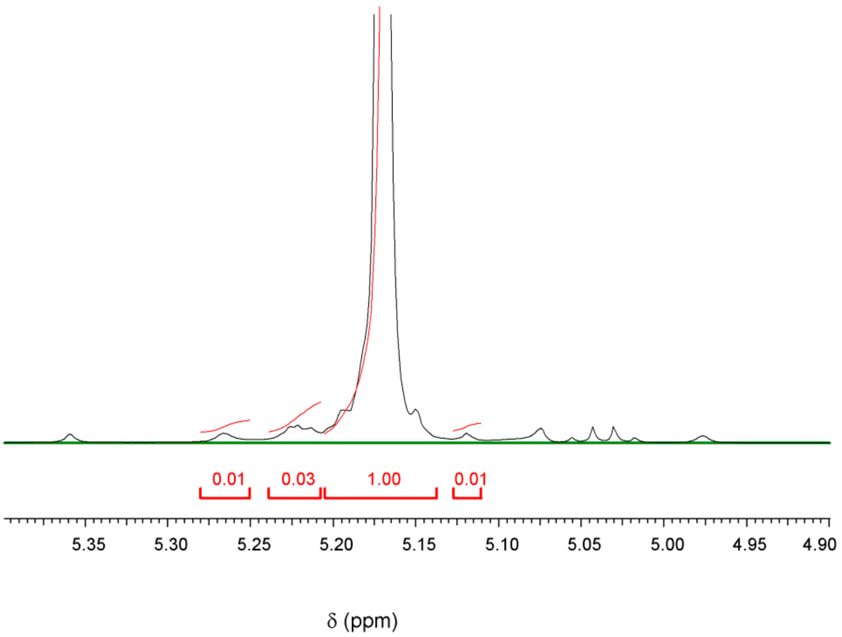

Figure S17. Tacticity of perdeuterated poly-D-lactic acid (**6**) (Table 1, entry 6) determined by ^{1H}{^{1}H} NMR spectroscopy.

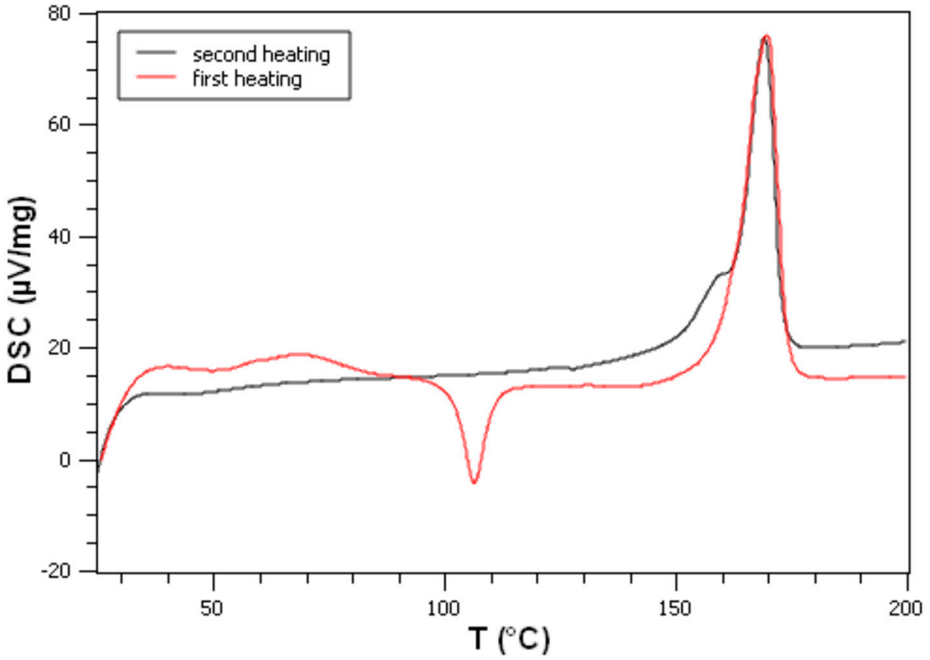

Figure S18. Differential-scanning calorimetry of perdeuterated poly-D-lactic acid (**6**) (Table 1, entry 6).

Sodium pyruvate-d₃ (1)

^{13}C NMR (100 MHz, $CDCl_3$) δ 25.7 (multiplet), 170.1 (s), 205.2 (s). HRMS: $C_3D_3O_3^-$ calculated: 90.0271; found: 90.0267; 91% D by comparison of isotopologue ratios.

D-Lactic acid-d₄ (2)

$[\alpha]_D^{20}$: +12.1° (c 2.05 in DCM) mg/mL

^{1}H NMR (400 MHz, D_2O) δ 1.39 (br s, residual).

^{13}C NMR (100 MHz, D_2O) δ 18.5 (m), 65.8 (m), 178.4 (s).

HRMS: $C_3D_4H_2O_3^-$ calculated: 93.0490; found: 93.0489; 98% D.

L-Lactic acid-d₄ (3)

$[\alpha]_D^{20}$: -12.3° (c 2.21 in DCM)

^{1}H NMR (400 MHz, D_2O) δ 1.32 (br s, residual).

^{13}C NMR (100 MHz, D_2O) δ 18.5 (m), 65.8 (m), 178.4 (s).

HRMS: $C_3D_4H_2O_3^-$ calculated: 93.0490; found: 93.0487; 94% D.

(D,D)-lactide (5)

^{2}H NMR (61 MHz, $CDCl_3$) δ 1.64 (br s, 3 D), 5.03 (br s, 1 D).

$[\alpha]_D^{20}$: +257.5 (c 1.65 in DCM)
